# An advanced bionic knee joint mechanism with neural network controller

**DOI:** 10.3389/fnbot.2023.1178006

**Published:** 2023-05-05

**Authors:** Changxian Xu, Zhongbo Sun, Chen Wang, Xiujun Wu, Binglin Li, Liming Zhao

**Affiliations:** Department of Control Engineering, Changchun University of Technology, Changchun, China

**Keywords:** tensegrity, bionic knee joint, kinematics, dynamics, noise-tolerant zeroing neural network model

## Abstract

In this article, a tensegrity-based knee mechanism is studied for developing a high-efficiency rehabilitation knee exoskeleton. Moreover, the kinematics and dynamics models of the knee mechanism are explored for bringing about further improvement in controller design. In addition, to estimate the performance of the bionic knee joint, based on the limit function of knee patella, the limit position functionality of the bionic knee joint is developed for enhancing the bionic property. Furthermore, to eliminate the noise item and other disturbances that are constantly generated in the rehabilitation process, a noise-tolerant zeroing neural network (NTZNN) algorithm is utilized to establish the controller. This indicates that the controller shows an anti-noise performance; hence, it is quite unique from other bionic knee mechanism controllers. Eventually, the anti-noise performance and the calculation of the precision of the NTZNN controller are verified through several simulation and contrast results.

## 1. Introduction

Rigid–flexible coupling robot technology has broad application prospects in medical diagnosis, pipeline fault detection, bionic structure manufacturing, and other fields. The tensegrity structure is an important part of this technology because of its lightweight and deployable characteristics. In the process of rehabilitation training, due to the symptoms of hemiplegia caused by stroke or cerebral hemorrhage in the patient, the rehabilitation training of the human knee joint becomes quite important. The knee joint can be regarded as a strongly coupled structure that is composed of bones, muscles, and ligaments. Hence, the components of a knee joint cannot be simply mapped to the traditional rigid linkage structure. More importantly, the motion characteristics of the knee joint should be analyzed when the movement takes place (Oshkour et al., 2011). Therefore, a bionic knee joint structure based on the principle of bionics can be constructed using the rigid–flexible coupling tensegrity structure.

The lower limb rehabilitation training of several rehabilitation robots has been analyzed in Arsenault and Gosselin (2005, 2006a,b, 2009); Vasquez and Correa (2007), Murray et al. (2015), Esquenazi and Talaty (2019), Nicholson-Smith et al. (2020), and Muralidharan and Wenger (2021). Yet, these robots have not been analyzed from the perspective of bionics. Since the tensegrity structure is considered to be a rigid–flexible coupling mechanism in Jung et al. (2018) and Liu et al. (2020), the problem has been considered from the viewpoint of bionics mechanism, but the dynamics analysis has not been carried out due to structural complexity. In Collins et al. (2015), Sankai and Sakurai (2018), Fitzsimons et al. (2019), and Kim et al. (2020), the wearable exoskeletons, which can be utilized for the patient rehabilitation process with upper and lower limb disabilities, have been established. Two bionic robots based on the ankle joint and the knee joint have been studied in Sun et al. (2019) and Zhang et al. (2020). These two bionic robots have been formatted as the ankle and knee joint tensegrity structures based on the human body constitution. However, owing to structural complexity, the dynamics models are not studied on a temporary basis. Therefore, when faced with a complex environment, these two bionic tensegrity structures may not meet the practical requirement. For the purpose of implementing the actual rehabilitation training scenario, the interference caused by external environments and patients, such as the mechanical manufacturing errors and the static friction between the rehabilitation robot with patients, cannot be avoided. As a result, the bionic tensegrity structure based on the dynamics analysis of human lower limb joints under noise environment is of great significance for further research of bionic human joints.

Considering the fact that during the human lower limb rehabilitation process, the torque, which is produced by the knee joint, cannot be ignored. In the different rehabilitation processes, the knee joint produces different knee torques. These knee torques should be considered in the design of dynamics models, which can demonstrate the influence of human knee forces on the bionic knee mechanism during the movement (Rifai et al., 2013, 2016). In addition, noise is unavoidable in the process of a bionic knee joint movement. In the field of anti-noise algorithm, the NTZNN algorithm has shown its advantages in the parallelly distributed computing and anti-noise fields (Hehne, 1990; Jin et al., 2017, 2018; Sun et al., 2020; Shi et al., 2021; Wei et al., 2021). In this article, the error caused by the actual trajectory and the desired trajectory can be seen as a non-linear objective function. Furthermore, the kinematics and dynamics of the tensegrity mechanism are studied. In addition, the limited function of the knee is realized by the mechanical design, for the purpose of showing the bionic performance of a knee joint tensegrity structure. The article is formulated as follows. In Section 2, it describes the structure of the human knee joint and the establishment process of the bionic knee joint tensegrity structure mapping model. The kinematics of the proposed structural mechanism are presented in Section 3. The dynamics model and the description of the NTZNN controller are proposed in Section 4. Simulation results in Section 5 prove that the bionic knee joint tensegrity structure is effective under the noise condition. Finally, in Section 6, the conclusion and future study are discussed. At the end of this paragraph, the main contributions of the article are summarized as follows.

1. A bionic knee joint tensegrity mechanism is proposed and studied. Furthermore, the limit position functionality of the knee joint is achieved through a mechanical design. In addition, the NTZNN model has shown its efficiency in designing a controller with the distractions of noise items.
2. A series of simulation and contrast results with the proportional integral differential (PID) controller are presented to prove the accuracy, computational efficiency, and the anti-noise performance of the NTZNN controller.

## 2. Establishment process of a bionic knee joint

In this section, by analyzing the muscles, bones, and ligaments of a knee joint, the tissues of a knee joint are simplified into one component that has the same function during the movement. In addition, a bionic knee joint structure based on the tensegrity structure is established according to the characteristics of a human lower limb. Based on the principle of bionics, the physical characteristics of the bionic knee joint, such as the limit self-locking function and muscle elasticity coefficient, are considered in the design process of a bionic knee joint.

### 2.1. Structural description of the knee joint

To establish the bionic knee joint mapping model, there is demand to investigate the structure of the human knee joint in detail. Therefore, in this subsection, the human knee joint is analyzed for further research. It is crucial to notice that only sagittal motions are considered in this article. Hence, the use of a human knee joint is mainly employed in the sagittal plane of the lower limb movement, such as going up- and downstairs, squatting, and jumping.

**Figure 1:**
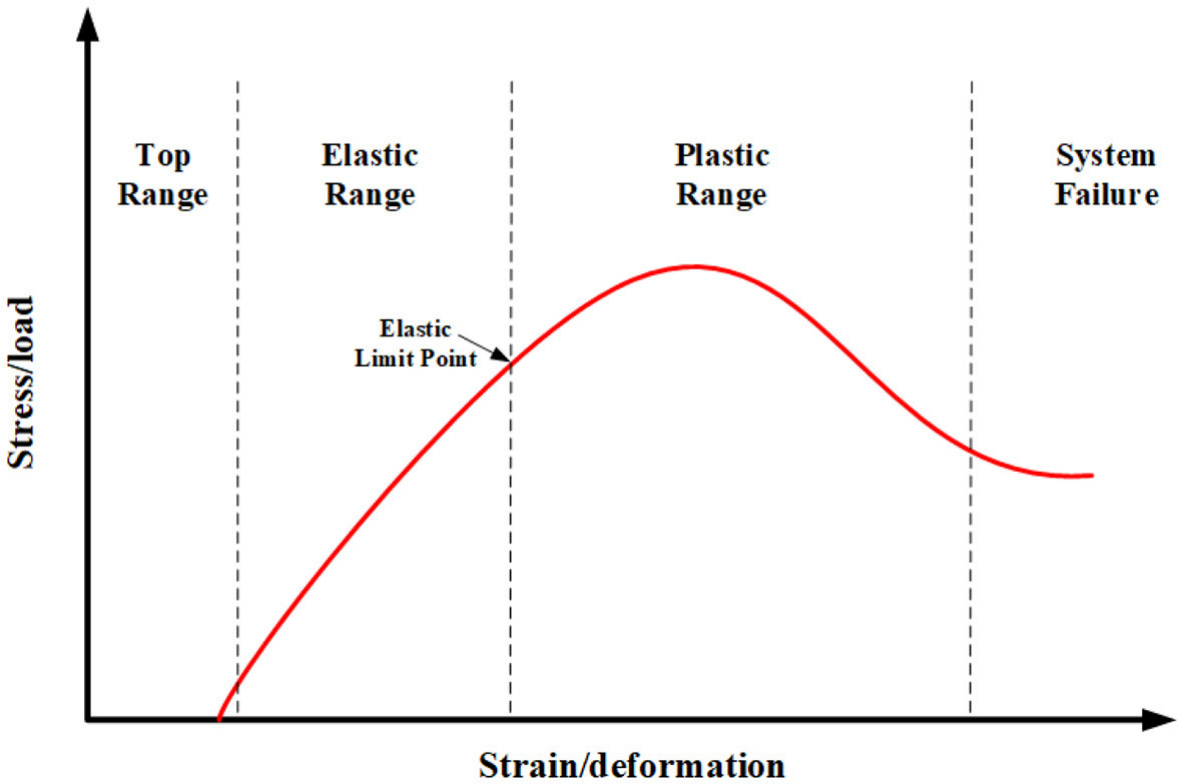
The relationship between the knee joint deformation with an external load (Bahr and Maehlum, 2003).

The knee includes four bones, the lower part of the femur, the upper part of the tibia, the upper part of the fibula, and the patella. Femur, tibia, and fibula act as weight bearing bones and force transfer during the lower limb movement. In addition, the patella plays a limiting role in preventing the lower limb from overextending during movement, thus avoiding injury to the human body. Therefore, the patella location-restricted self-locking function is the key function of bionic joint tensegrity. Furthermore, due to the knee bearing the responsibility of supporting the body weight, its stiffness is higher. Thus, the skeleton of the knee joint can be regarded as the strut of a tensegrity structure, which indicates that the stiffness of a strut is infinite compared with the cable. The muscles and ligaments of the human lower limb are responsible for generating and transferring the load. Knee muscles can be divided into four groups according to their role in the lower limb movement. More importantly, the deformation of a muscle relative to the external load is shown in Figure 1. The muscle viscoelastic coefficient is similar to the spring damping coefficient, which should be considered in the stage of elastic range. To a certain degree, the bionics performance of the knee tensegrity mechanism can be realized by considering the viscoelastic coefficient.

### 2.2. Establishment process of the human knee joint mapping model

The knee joint mapping model and the bionic tensegrity structure are constructed in this subsection. For reducing the human tissue structure into a low-degree-of-freedom tensegrity structure, the strategy is to simplify the knee with basically the same function into the one structure. Under this strategy, the hamstring and tibialis anterior muscles are reduced to one muscle. The sartorius, semimembranosus, gracilis, and semitendinosus can be seen as one muscle. Furthermore, the quadriceps is simplified to a muscle. In addition, the gastrocnemius could be thought of as a muscle. As regards the bone and the bone-like tissue, the fibula and tibia are decreased to a single bone for the reason of their similar functionality. Due to their peculiar function, the patella and tissues that perform the same function are simplified into two struts. The bionic patellar groove, which can also be called as the pulley groove, is established to implement the ultimate self-locking function of the knee joint. A limiting device is constructed on the bionic patella groove. It prevents the pulley from going off course as it slides through the bionic patellar groove. The self-locking function of the bionic knee joint is realized through the aforementioned mechanism design ultimately. As regards the bionic knee joint structure, the rotating pair and the first strut can be seen as the simplified bionic patella structures. For the sake of simplifying the complexity of a bionic patella mechanism, we should also ensure that the bionic mechanism should realize the bionic purpose. The rotation pair should be fixed for limiting the bionic knee extension movement under the action of external forces.

## 3. Analyses of the bionic knee joint

### 3.1. Description of a bionic knee joint mechanism

From the viewpoint of bionics, the patella and similar functional tissues perform two main biological functions during locomotion. In the first place, it distributes the pressure more widely over the femur, with the strategy of increasing the contact range between the patellar tendon and the patella. In the second place, it helps in knee extension by creating a forward displacement of the quadriceps tendon throughout the range of motion. However, the range of motion of the patella is fairly small, relative to the overall motion of the knee joint from full flexion to full extension (Hehne, 1990). Furthermore, when the knee joint moves, the displacement of the patella leading edge is not obvious when compared with the femur, fibula, and tibia. As a result, its primary function is to protect the quadriceps femoral tendon. For the sake of simplifying the complexity of the bionic knee structure, which also reduces the degree of structural freedom, as a result, it is convenient to analyze the dynamics model of the bionic knee joint structure in the next step. Moreover, for achieving the self-locking function of the patella and for the purpose of preventing knee hyperextension, the revolute joint pair is fixed to the bionic patella. The pulley is slid in the bionic patella groove, which is aimed to finish the self-locking function of the knee joint during the lower limb movement. The range of knee flexion angles for a healthy adult is approximately 130^*o*^ to 140^*o*^, but the stroke patients cannot complete the entire motion range. However, the range of motion of the affected limb is increased when the affected limb's physical condition is improved during the rehabilitation process. Therefore, the length of a bionic patellar groove can be changed to satisfy the different rehabilitation training stages.

### 3.2. Singular configuration

Singular configuration refers to the case where degeneracy occurs between the input and output variables of the structure (Arsenault and Gosselin, 2005). However, due to the limiting properties provided by the strut *CF*, *DE*, and bionic patellar groove, the bionic knee tensegrity structure may stop moving before reaching the singular configuration. Thus, the singular configuration is reached when the knee extension is the upper working boundary of the tensegrity structure. However, when the movement of the mechanism takes place, this situation should be avoided. In this case, the tensegrity system is degenerated, which may cause the tensegrity system to collapse. Furthermore, the situation is similar to the undue knee joint movements that could happen in real life rehabilitation.

### 3.3. Working curve

In the design process of a tensegrity structure, it is very important to study the working curve of the mechanism. In this subsection, the working curves of nodes *C* and *D* are obtained through the ADAMS software kinematic simulation, which can get the working spaces of angles θ and γ. Since two sets of linkage mechanisms are axially symmetric about the *y* axis, the operating curves of the two nodes are identical. In the kinematic simulation, the external forces are perpendicular to the *x* axis, which are acting on the nodes *C* and *D*; hence, the operating curve goes from the initial self-equilibrium state to the limit position when θ is equal to 90^*o*^. It can be seen from the kinematic simulation that the limiting mechanism based on the principle of bionics can prevent the overextension of the bionic knee joint structure under the action of external forces on nodes *C* and *D*. However, the displacements of points *A* and *B* cannot be restricted through the two-link mechanism alone. It reflects the significance for the bionic patellar groove's constraint functionality when facing the movement at points *A* and *B*. Furthermore, the *y*_*C*_ and *y*_*D*_ decrease when the force direction is opposite to the previous situation, the circumstance corresponds to the knee flexion. The *y*_*C*_ and *y*_*D*_ will decrease to zero eventually, yet the situation should be avoided in the actual operating circumstance.

## 4. Dynamics model and controller

To exploit the efficiency of the bionic knee joint tensegrity structure in the rehabilitation process, the dynamics model and the NTZNN controller of the presented tensegrity bionic knee joint are developed and studied in this section.

### 4.1. Dynamics model

#### 4.1.1. Hypotheses

The following hypotheses are proposed to derive the dynamics model of the tensegrity structure:

1. The gravitational potential energy is neglected for the purpose of reducing the dynamics model's complexity.
2. The springs are massless.
3. Each strut is a thin rod of *w* mass and the moment of inertia is $\frac{1}{12}wB^{2}$.
4. The spring is linearly damped with coefficients *a*_1_, *a*_2_, *a*_3_, and *a*_4_, in which *a*_1_ is equal to *a*_2_.

#### 4.1.2. Equation form of the Lagrangian approach

As regards the tensegrity structure, it has two degrees of freedom, therefore, the β and γ are selected as the generalized coordinates. The dynamics model is developed by utilizing the Lagrangian approach, which is defined by


(1) $$\frac{\text{d}}{\text{d}t}\frac{\partial K}{\partial \dot{\text{q}}}-\frac{\partial K}{\partial \text{q}}+\frac{\partial P}{\partial \text{q}}=\text{f},$$

where *P* and *K* express the potential and kinetic energies of the tensegrity structure, $\text{f}={\left[f_{1},f_{2}\right]}^{\text{T}}$ is the non-conservative force, and the **q** is equal to [β, γ]^T^. To reflect the influence on the dynamics model of viscoelasticity caused by muscle deformation, the non-conservative forces are formed in this subsection. The non-conservative forces correspond to the damping forces in the springs. In the tensegrity mechanism, the kinetic energy of the system is generated by the movement of the strut alone, thus the kinetic energy can be formatted as


(2) $$\begin{array}{l} \begin{array}{l} K=w_{1}B_{2}^{2}{\overset{•}{\beta }}^{2}+\frac{1}{3}w_{1}B_{1}^{2}{\left(\overset{•}{\beta }+\overset{•}{\gamma }\right)}^{2}+w_{1}B_{1}B_{2}cos\gamma \overset{•}{\beta }(\overset{•}{\beta }+\overset{•}{\gamma })+ \end{array}\\ \begin{array}{l} \frac{1}{3}w_{2}B_{2}^{2}{\overset{•}{\beta }}^{2}, \end{array} \end{array}$$

where *w*_1_ and *w*_2_ are the masses of struts *B*_1_ and *B*_2_, individually.

In addition, the potential energy could be defined as:


(3) $$\begin{array}{ll} P= & k_{1}{\left(\sqrt{{\left(B_{1}\cos\alpha -2(B_{3}+B_{2}\cos\beta )\right)}^{2}+{\left(B_{2}\sin\beta \right)}^{2}}-z_{01}\right)}^{2}+\\ & \frac{1}{2}k_{3}{\left(2(B_{3}+B_{2}\cos\beta )-z_{03}\right)}^{2}\;\\ & +\frac{1}{2}k_{4}{\left(2(B_{1}\cos\alpha -(B_{3}+B_{2}\cos\beta ))-z_{04}\right)}^{2}, \end{array}$$

where the subentry potential energy *P*_1_ is equal to *P*_2_. The *z*_01_, *z*_03_, and *z*_04_ are the initial lengths of the springs. The non-conservative force caused by spring damping is expressed as


(4) $$\begin{array}{l} \begin{array}{l} f_{1}=-2c_{1}{ż}_{1}\frac{\partial z_{1}}{\partial \beta }-c_{3}{ż}_{3}\frac{\partial z_{3}}{\partial \beta }-c_{4}{ż}_{4}\frac{\partial z_{4}}{\partial \beta } \end{array}\\ \begin{array}{l} f_{2}=-2c_{1}{ż}_{1}\frac{\partial z_{1}}{\partial \gamma }-c_{3}{ż}_{3}\frac{\partial z_{3}}{\partial \gamma }-c_{4}{ż}_{4}\frac{\partial z_{4}}{\partial \gamma }, \end{array} \end{array}$$

where the *z*_1_, *z*_3_, and *z*_4_ are the presented lengths of the springs. As shown in Figure 1, the coefficient of muscle elasticity in the elastic range is similar to the coefficient of spring damping. The muscles are similar to the springs in the bionic knee joint. When muscles are deformed, the resistance produced by the friction between muscle fibers sticks to the extension and contraction of muscles. Consequently, the bionic performance of the bionic knee joint structure can be achieved by considering the elastic damping in the dynamics modeling process.

Hence, the dynamics model can be formatted as follows:


(5) $$\begin{array}{l} \text{M}\overset{..}{\text{q}}+\text{H}{\dot{\text{q}}}_{qh}+\text{G}{\dot{\text{q}}}_{qg}+\text{C}\dot{\text{q}}+\text{T}+\text{u}+{\text{τ}}_{R}=0, \end{array}$$

where ${\overset{•}{\text{q}}}_{qh}$=${\left[{\overset{•}{\beta }}^{2},{\overset{•}{\gamma }}^{2}\right]}^{\text{T}}$, ${\overset{•}{\text{q}}}_{qg}$=${\left[\overset{•}{\beta }\overset{•}{\gamma },\overset{•}{\gamma }\overset{•}{\beta }\right]}^{\text{T}}$, ***τ***_*R*_, and **u** are the knee torque and control law, the matrix **C** has relations with the non-conservative force, and $\text{T}={\left[T_{1},T_{2}\right]}^{\text{T}}$ is the matrix that is associated with the potential energy.

Detailedly,


(6) $$\begin{array}{l} W_{1,1}=2w_{1}B_{1}^{2}+\frac{2}{3}w_{1}B_{2}^{2}+\frac{2}{3}w_{2}B_{2}^{2}+2w_{1}B_{1}B_{2}\cos\gamma , \end{array}$$

(7) $$\begin{array}{l} W_{1,2}=W_{2,1}=\frac{2}{3}w_{1}B_{1}^{2}+w_{1}B_{1}B_{2}\cos\gamma , \end{array}$$

(8) $$\begin{array}{l} W_{2,2}=\frac{2}{3}w_{1}B_{1}^{2}, \end{array}$$

moreover,


(9) $$\begin{array}{l} \text{H}=\left[\begin{array}{cc} 0 & -w_{1}B_{1}B_{2}\sin\gamma \\ w_{1}B_{1}B_{2}\sin\gamma & 0 \end{array}\right], \end{array}$$

and,


(10) $$\begin{array}{l} \text{G}=\left[\begin{array}{cc} -2w_{1}B_{1}B_{2}\sin\gamma & 0\\ 0 & 0 \end{array}\right]. \end{array}$$

### 4.2. NTZNN controller

A continuous-time NTZNN model is utilized to design the control law in this subsection. In the process of the operation of the mechanism, the noises, which may include mechanical structure error, mechanical vibration, friction between components, feedback signal noise, external static friction and other factors, are inevitable items. In addition, the knee torque, which is generated by the human knee during rehabilitation, should be considered in the dynamics modeling process. In the human lower limb recovery process, different lower limb rehabilitation stages may cause different torques which are produced by the knee. For example, in the early stage of rehabilitation training, lower limb hemiplegia that is caused by stroke and other diseases may lead to an uncoordinated movement of lower limbs, which make lower limbs unable to move according to the patient's real intention. The actual lower limb movement trajectory may be in conflict with the rehabilitation robot. In addition, there is a special rehabilitation stage, which corresponds to be deprived of the nerve conduction function between the patient's central nervous system and the lower limb skeletal muscles. It could also be considered as the passive rehabilitation stage of a patient who has received a lower limb joint surgery or a total knee replacement, and in these circumstances, the knee torque ***τ***_*R*_ is very small when compared with other situations (Cao and Huang, 2020). Therefore, in the modeling process, the knee torque could not be overlooked, and the knee torque ***τ***_*R*_ should be considered in the modeling process. The knee torque ***τ***_*R*_ could be seen as a constant torque, for the reason that in the same rehabilitation stage, the knee torque is roughly the same.

In this subsection, the problem is formatted as:


(11) $$\begin{array}{l} \phi \left(y\left(p\right)\right)=0\in \mathbb{R},p\in \left[0,+\infty \right), \end{array}$$

furthermore,


(12) $$\begin{array}{l} \frac{\text{d}\phi (y(p))}{\text{d}p}=\frac{\partial \phi (y(p))}{\partial t}+\frac{\partial \phi (y(p))}{\partial y(p)}\frac{\text{d}y(p)}{dp}={\dot{\phi }}_{p}(y(p))+\\ \text{R}(y(p))\frac{\text{d}y(p)}{\text{d}p}, \end{array}$$

where **R**(*y*(*p*)) is equal to ∂***ϕ***(*y*(*p*))/*∂y*(*p*).

An error function can be generalized as:


(13) $$\begin{array}{l} \text{e}(p)=0-\phi (y(p)). \end{array}$$

Hence, a noise-suppressing zeroing dynamics model is defined by:


(14) $$\begin{array}{l} \dot{\text{e}}(p)=-\beta \text{e}(p)-\lambda \int_{0}^{t}e(\delta )\text{d}\delta , \end{array}$$

where β and λ are positive constants. δ is the time interval. Eventually, a continuous-time NTZNN model which is polluted by noise is given as:


(15) $$\begin{array}{l} \dot{y}(p)=-{\text{R}}^{-1}(y(p))(\beta \phi (y(p))+{\dot{\phi }}_{p}(y(p))+\lambda \int_{0}^{t}\phi (y(\delta ))\text{d}\delta +\text{ε}(p)), \end{array}$$

which ***ε***(*p*) is the noise item. In this subsection, considering the influence of noises and knee joint torques on the bionic knee joint control algorithm, an anti-noise ZNN model is established as the control algorithm to control the bionic knee joint dynamics model. To further study the NTZNN model, the theories are presented as follows.

**Theorem 1**. The ϕ(*p*) can be seen as a vector, which is to say that the time-varying vector Ξ(*p*) can be managed through utilization of the NTZNN model global convergence from selecting the initial states (Ξ_0_≠0∈ℝ) to the theoretical solution $\hat{\Xi }\left(p\right)$ randomly with constant noise ($R\left(p\right)=\tilde{R}\in \mathbb{R}$).

**Proof** The noise-polluted NTZNN model could be transformed based on the Laplace transformation, which can be formatted as follows


$$\begin{array}{l} jy\left(j\right)-y\left(0\right)=-\Lambda y\left(j\right)-\frac{\iota }{j}y\left(j\right)+R\left(j\right). \end{array}$$

As a result, the equation could be formed as


(16) $$\begin{array}{l} y\left(j\right)=\frac{j\left[y\left(0\right)+R\left(j\right)\right]}{j^{2}+j\Lambda +\iota }. \end{array}$$

Furthermore, the transfer function of equation (16) should be formatted as *j*/(*j*^2^+*jΛ*+ι). In addition, the $j_{1}=\left(-\Lambda +\sqrt{\Lambda^{2}-4\iota }\right)/2$ and $j_{2}=\left(-\Lambda -\sqrt{\Lambda^{2}-4\iota }\right)/2$ are poles of the transfer function. Moreover, on account of Λ>0 and ι>0, the poles of the transfer function lie in the left half-plane, which can testify that the time-varying problem, which is polluted with the constant noise *R*(*p*), is stable. In addition, for the reason of the noise item is constant, hence, $R\left(j\right)=\tilde{R}/j$. In summary, the following result can be defined as


$$\begin{array}{l} \underset{p\to \infty }{\lim}y\left(p\right)=\underset{s\to 0}{\lim}jy\left(j\right)=\underset{j\to 0}{\lim}\frac{j^{2}\left[y\left(0\right)+\frac{\tilde{R}}{j}\right]}{j^{2}+j\Lambda +\iota }=0. \end{array}$$

The proof is thus complete.

**Theorem 2**. When ϕ(*p*) can be seen as a vector, it is to say that the time-varying vector Ξ(*p*) can be managed through utilization of the NTZNN model global convergence from selecting the initial states (Ξ_0_≠0∈ℝ) to the solution $\hat{\Xi }\left(p\right)$ with linear noise ($R\left(p\right)=p\tilde{R}\in \mathbb{R}$).

**Proof** For the reason of the Laplace transformation, the NTZNN model with linear noise polluted ($R\left(p\right)=t\tilde{R}$) should be defined as


(17) $$\begin{array}{l} jy\left(j\right)=y\left(0\right)-\Lambda y\left(j\right)-\frac{\iota }{j}y\left(j\right)+\frac{\tilde{R}}{j^{2}}, \end{array}$$

where $\tilde{R}/j^{2}$ is the Laplace transformation of *R*(*p*). Hence, the following results could be formatted through investigation of the final value theorem


$$\begin{array}{l} \underset{t\to \infty }{\lim}y\left(p\right)=\underset{j\to 0}{\lim}\frac{j^{2}\left[y\left(0\right)+\frac{\tilde{R}}{j^{2}}\right]}{j^{2}+j\Lambda +\iota }=\frac{\tilde{R}}{\iota }. \end{array}$$

As a result, a conclusion that $\underset{p\to \infty }{\lim}y\left(p\right)\to 0$ with ι → ∞ could be drawn. The proof is complete.

## 5. Experiments and analysis

In this section, through the experiments, the effectiveness of the bionic knee joint is verified under the interference of noise items.

### 5.1. The performance of a bionic knee joint tensegrity structure in different stages of rehabilitation

The moment and fixed noise of the knee joint in different rehabilitation stages are considered in the experiment to prove the anti-noise performance of the NTZNN controller and the accuracy of the bionic knee joint dynamics model. In the said experiments, three kinds of knee torques are proposed to represent the forces that are generated in different recovery stages. The three stages, namely, resistance rehabilitation stage, auxiliary rehabilitation stage, and passive rehabilitation stage, are distinguished by the knee joint torques, which are −40, 150, and 0 *N*·*m*, respectively (Zhao and Xu, 2011). Furthermore, to reflect the superiority of the NTZNN algorithm in an anti-noise field, a kind of mixed noise, which is formed by constant noise, linear noise, and random noise is presented in this subsection. The fixed noise is defined by


(18) $$\begin{array}{l} \varepsilon \left(t\right)=\eta +\kappa t+\mu \left(t\right), \end{array}$$

in which ***η*** is the constant noise, ***κ****t* is the linear noise, and ***μ***(*t*) is the random noise. ***η***, ***κ***, and ***μ*** are the coefficients.

The desired trajectory of the bionic knee joint in the experiment is acquired and fitted by ADAMS software. Therefore, the desired trajectory can delegate the real motion trajectory of the bionic knee joint in rehabilitation training. The motion trajectory can be defined as follows


(19) $$\begin{array}{l} \theta_{d}=1.33-0.2186\times \cos\left(0.03749t\right)-0.007953\times \sin\left(0.03749t\right), \end{array}$$

(20) $$\begin{array}{l} \begin{array}{ll} \gamma_{d} & =3.159-0.4651\times \cos(0.03748t)-0.01676\times \sin(0.03748t) \end{array}\\ \;\begin{array}{l} -\;0.1284\times \cos(0.07496t)-0.008771\times \sin(0.07496t). \end{array} \end{array}$$

#### 5.1.1. Resistance rehabilitation stage

**Figure 2:**
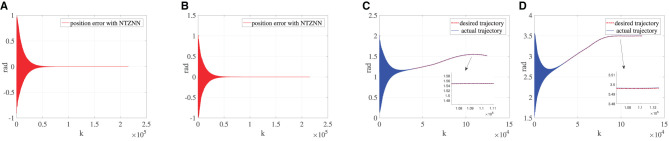
Under the circumstance of ***τ***_*R*_ is equal being −40, **(A)** is the error between the desired trajectory and the actual trajectory of θ, **(B)** the error between the desired trajectory and the actual trajectory of γ, **(C)** the desired the trajectory and the actual trajectory of θ and **(D)** the desired trajectory and the actual trajectory of γ.

It is assumed that the patient is in the resistance rehabilitation stage. Although the patient can move the affected limb, it cannot carry out a series of rehabilitation activities completely according to the patient's real movement intention. The actual movement trajectory of the affected limb may encounter human–machine confrontation with the rehabilitation robot due to the uncoordinated movement of the affected limb. In addition, a series of rehabilitation training actions cannot be repeated for a long time due to muscle atrophy of the affected limb and various other reasons. Therefore, in the resistance rehabilitation stage, the knee joint torque generated by the affected knee joint is defined as a negative value, where the knee torque ***τ***_*R*_ is equal to -40 *N*·*m*. The desired trajectory is utilized to explore the performance of the NTZNN controller. Figure 2 shows the position error between the actual trajectory and the desired trajectory of the bionic knee joint angle when using the NTZNN controller. The actual trajectory can converge to the desired trajectory using the NTZNN controller rapidly. The interference of internal and external noises to the model is considered during the design process of the NTZNN controller. The experimental results show that the fixed noise can be suppressed using the NTZNN model, which proves that the NTZNN algorithm has strong robustness and noise suppression ability. Although at the initial stage, there is an oscillation between the expected trajectory and the actual trajectory, nevertheless, with the increase in iterations, the error between the desired trajectory and the actual trajectory decreases and could reach the level of 1 × 10^−4^ gradually.

#### 5.1.2. Assistance rehabilitation stage

**Figure 3:**
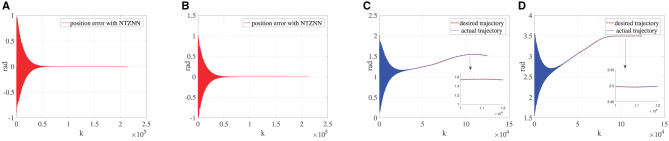
Under the circumstance of ***τ***_*R*_ being equal to 150, **(A)** is the error between the desired trajectory and the actual trajectory of θ, **(B)** the error between desired trajectory and the actual trajectory of γ, **(C)** the desired trajectory and the actual trajectory of θ and **(D)** the desired trajectory and the actual trajectory of γ.

The physical condition of the affected limb will improve after a period of rehabilitation training. In this process, the affected limb of the patient moves smoothly, but the affected limb is generally unable to produce enough torque to carry out rehabilitation training in accordance with the requirements of rehabilitation training. Hence, patients still need the bionic knee to provide an additional torque to assist the affected limb to complete rehabilitation training in the assistance rehabilitation phase. The knee torque that a healthy adult can produce is around 170 *N*·*m* to 300 *N*·*m*. Although the affected limb can produce more torques in the auxiliary rehabilitation stage, it is still smaller than the normal torque. Thus, the knee joint torque is set as 150 *N*·*m* in the assistance rehabilitation stage. As shown in Figure 3, the fixed noise and knee torque 150 *N*·*m* are taken into account in the designing process of the NTZNN controller. The experimental results show that the NTZNN model could suppress the noise available, which makes the controller to be provided with robustness and anti-noise performances. In the assistance rehabilitation stage, the main objective for the controller is to manage the bionic knee movement under noise pollution. In addition, the purposes for using a bionic knee are to enhance the muscle strength by assisting with rehabilitation exercises and to facilitate the reconstruction of the somatosensory stimuli according to rehabilitation goals. The experiments have proved that under the noise pollution, the NTZNN controller could suppress the fixed noise and control the bionic knee to assist the patient to complete the rehabilitation goals, which demonstrate the accuracy and effectiveness of the NTZNN approach.

#### 5.1.3. Passive rehabilitation stage

**Figure 4:**
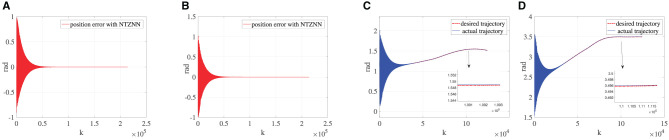
Under the circumstance of ***τ***_*R*_ being equal to 0, **(A)** is the error between the desired trajectory and the actual trajectory of θ, **(B)** the error between the desired trajectory and the actual trajectory of γ, **(C)** the desired trajectory and the actual trajectory of θ and **(D)** the desired trajectory and the actual trajectory of γ.

To verify the versatility of the bionic knee joint, that is, rehabilitation training can be completed under various circumstances, this subsection designs a passive rehabilitation stage. This situation applies to patients who have undergone knee surgery or total knee replacement surgery. Therefore, the rehabilitation training action of the affected limb is completely driven by the bionic knee joint. Compared with the torque generated in other rehabilitation stages, the knee joint torque in the passive rehabilitation stage is very small, so the knee joint torque is approximately equal to zero. Since the knee joint torque is zero, the only interference in the dynamics model is the fixed noise during passive rehabilitation. As shown in Figure 4, the dynamics model can achieve a low error and the convergence speed is faster than other rehabilitation stages under the control of the NTZNN algorithm. It also verifies that the knee torque ***τ***_*R*_ should be seen as a disturbance torque during the movement of the bionic knee joint, which demonstrates the importance of the NTZNN controller's anti-noise performance.

### 5.2. Contrast experiments

**Figure 5:**
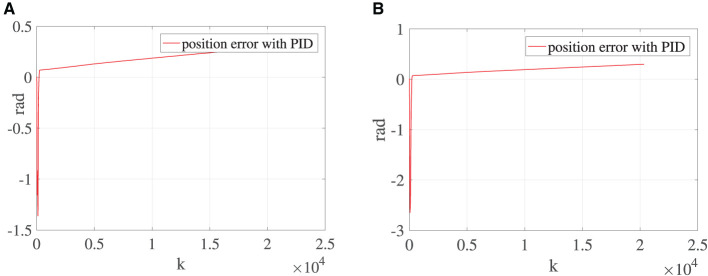
Under the circumstance of ***τ***_*R*_ being equal to 150, **(A)** is the error between the desired trajectory and the actual trajectory of θ and **(B)** the error between the desired trajectory and the actual trajectory of γ.

To demonstrate the superiority of the NTZNN algorithm in the field of noise suppression, the PID algorithm is used as the controller to control the bionic knee joint dynamics model in the contrast experiments under the noise condition. In the actual rehabilitation training process, not only will the external environment cause interference to the rehabilitation training process, but the patient's own health conditions will also cause certain interference to the rehabilitation training process, such as an involuntary spasm of the affected limb. The experimental results show that, the position errors of bionic knee joint angles θ and γ will increase with the introduction of fixed noise and knee torque gradually. It could be seen from Figure 5 that, with the growing number of iterations, the position error between the desired trajectory and the actual trajectory increases to the extent that it can affect the operation of the bionic knee joint. The interferences of external environment and patients to the rehabilitation training process are inevitable in the actual training situation under the interference of non-ideal factors. If the interferences to a rehabilitation training process are ignored when designing the control algorithm of a bionic knee joint, it may cause secondary injury to the affected limb during the rehabilitation process. Therefore, the NTZNN algorithm with an anti-noise ability offers great advantages in the design course of a bionic knee joint control algorithm. An NTZNN algorithm is established as the controller of a bionic knee joint dynamics model by analyzing the influence of the external noise and the knee joint torque on the bionic knee joint control algorithm in the actual rehabilitation training process. The experiments of three different rehabilitation stages and comparison experiments show that the NTZNN algorithm has significant advantages in suppressing non-ideal factors in rehabilitation training.

## 6. Conclusion

In this article, a bionic knee joint tensegrity structure in noise environment has been developed and studied from the viewpoint of principle of bionics. Moreover, the knee joint torques at different rehabilitation stages have been considered in the controller design process, so as to reflect the influence of the human knee force acting on the bionic knee joint tensegrity structure. The dynamics model of the bionic knee mechanism has been established by means of analyzing the kinetic energy and potential energy of the system. Eventually, the simulations and contrast results have shown that the NTZNN controller has advantages in noise suppression and computational efficiency. The main work in future would be to undertake research on the bionic hip joint structure with a remarkable bionic performance.

## Data availability statement

The original contributions presented in the study are included in the article/supplementary material, further inquiries can be directed to the corresponding author.

## Author contributions

CX and ZS: data curation and software. CX, ZS, and CW: conceptualization. CX, XW, BL, and LZ: methodology. XW, LZ, and CW: formal analysis. CX, CW, and XW: writing-original draft. ZS and LZ: supervision. ZS, CW, and LZ: validation. All authors have read and agreed to the published version of the manuscript.

## References

[B1] Arsenault M. Gosselin C. M. (2005). Kinematic, static, and dynamic analysis of a planar one-degree-of-freedom tensegrity mechanism. Trans. ASME 127, 1152–1160. 10.1115/1.1913705

[B2] Arsenault M. Gosselin C. M. (2006a). Kinematic, static and dynamic analysis of a planar 2-DOF tensegrity mechanism. Mech. Mach. Theory 41, 1072–1089. 10.1016/j.mechmachtheory.2005.10.014

[B3] Arsenault M. Gosselin C. M. (2006b). Kinematic, static, and dynamic analysis of a spatial three-degree-of-freedom tensegrity mechanism. J. Mech. Design 128, 1061–1069. 10.1115/1.2218881

[B4] Arsenault M. Gosselin C. M. (2009). Kinematic and static analysis of a 3-PUPS spatial tensegrity mechanism. Mech. Mach. Theory 44, 162–179. 10.1016/j.mechmachtheory.2008.02.005

[B5] Bahr R. Maehlum S. (2003). C*linical Guide to Sports Injuries, Human Kinetics*.

[B6] Cao Y. Huang J. (2020). Neural-network-based nonlinear model predictive tracking control of a pneumatic muscle actuator-driven exoskeleton. IEEE/CAA J. Automat. Sin. 7, 1478–1488. 10.1109/JAS.2020.1003351

[B7] Collins S. H. Wiggin M. B. Sawicki G. S. (2015). Reducing the energy cost of human walking using an unpowered exoskeleton. Nature 522, 212–215. 10.1038/nature14288 25830889 PMC4481882

[B8] Esquenazi A. Talaty M. (2019). Robotics for lower limb rehabilitation. Phys. Med. Rehabil. Clin. N. Am. 30, 385–397. 10.1016/j.pmr.2018.12.012 30954154

[B9] Fitzsimons K. Acosta A. M. Dewald J. P. A. Murphey T. D. (2019). Ergodicity reveals assistance and learning from physical human-robot interaction. Sci. Robot. 4, 60–79. 10.1126/scirobotics.aav6079 31531410 PMC6748650

[B10] Hehne H. J. (1990). Biomechanics of the patellofemoral joint and its clinical relevance. Clin. Orthop. Relat. Res. 258, 73–85. 10.1097/00003086-199009000-00011 2394060

[B11] Jin L. Zhang Y. Li S. Zhang Y. (2017). Noise-tolerant ZNN models for solving time-varying zero-finding problems: a control-theoretic approach. IEEE Trans. Automat. Control 62, 992–997. 10.1109/TAC.2016.2566880

[B12] Jin L. Zhang Y. Qiu B. (2018). Neural network-based discrete-time Z-type model of high accuracy in noisy environments for solving dynamic system of linear equations. Neural Comput. Appl. 29, 1217–1232. 10.1007/s00521-016-2640-x

[B13] Jung E. Ly V. Cessna N. Ngo M. L. Castro D. SunSpiral V.. (2018). “Bio-inspired tensegrity flexural joints,” in 2018 IEEE International Conference on Robotics and Automation (Brisbane, QLD: IEEE), 5561–5566. 10.1109/ICRA.2018.846102

[B14] Kim K. Agogino A. K. Agogino A. M. (2020). Rolling locomotion of cable-driven soft spherical tensegrity robots. Soft Robot. 7, 346–361. 10.1089/soro.2019.0056 32031916 PMC7301328

[B15] Liu S. Li Q. Wang P. Guo F. (2020). Kinematic and static analysis of a novel tensegrity robot. Mech. Mach. Theory 149, 103788. 10.1016/j.mechmachtheory.2020.103788

[B16] Muralidharan V. Wenger P. (2021). Optimal design and comparative study of two antagonistically actuated tensegrity joints. Mech. Mach. Theory 159, 104249. 10.1016/j.mechmachtheory.2021.104249

[B17] Murray S. A. Ha K. H. Hartigan C. Goldfarb M. (2015). An assistive control approach for a lower-limb exoskeleton to facilitate recovery of walking following stroke. IEEE Trans. Neural Syst. Rehabil. Eng. 23, 441–449. 10.1109/TNSRE.2014.2346193 25134084

[B18] Nicholson-Smith C. Mehrabi V. Atashzar S. F. Patel R. V. (2020). A multi-functional lower- and upper-limb stroke rehabilitation robot. IEEE Trans. Med. Robot. Bionics 2, 549–552. 10.1109/TMRB.2020.3034497

[B19] Oshkour A. Osman N. A. Davoodi M. Bayat M. Yau Y. Abas W. W. (2011). “Knee joint stress analysis in standing,” in 5th Kuala Lumpur International Conference on Biomedical Engineering. p. 179–181. 10.1007/978-3-642-21729-6_47

[B20] Rifai H. Mohammed S. Djouani K. Amirat Y. (2016). Toward lower limbs functional rehabilitation through a knee-joint exoskeleton. IEEE Trans. Control Syst. Technol. 25, 1–8. 10.1109/TCST.2016.256538526068547

[B21] Rifai H. Mohammed S. Hassani W. Amirat Y. (2013). Nested saturation based control of an actuated knee joint orthosis. Mechatronics 23, 1141–1149. 10.1016/j.mechatronics.2013.09.007

[B22] Sankai Y. Sakurai T. (2018). Exoskeletal cyborg-type robot. Sci. Robot. 187, 1–9. 10.1126/scirobotics.aat3912 33141743

[B23] Shi T. Tian Y. Sun Z. Liu K. Jin L. Yu J. (2021). Noise-tolerant neural algorithm for online solving yang-baxter-type matrix equation in the presence of noises: a control-based method. Neurocomputing 424, 84–96. 10.1016/j.neucom.2020.10.110

[B24] Sun J. Song G. Chu J. Ren L. (2019). An adaptive bioinspired foot mechanism based on tensegrity structures. Soft Robot. 6, 778–789. 10.1089/soro.2018.0168 31414964

[B25] Sun Z. Shi T. Wei L. Sun Y. Liu K. Jin L. (2020). Noise-suppressing zeroing neural network for online solving time-varying nonlinear optimization problem: a control-based approach. Neural Comput. Appl. 32, 11505–11520. 10.1007/s00521-019-04639-2

[B26] Vasquez R. E. Correa J. C. (2007). “Kinematics, dynamics and control of a planar 3-DOF tensegrity robot manipulator,” in Proceedings of the ASME 2007 International Design Engineering Technical Conferences and Computers and Information in Engineering Conference. Volume 8: 31st Mechanisms and Robotics Conference, Parts A and B (Las Vegas, NV: ASME), 855–866. 10.1115/DETC2007-34975

[B27] Wei L. Jin L. Yang C. Chen K. Li W. (2021). New noise-tolerant neural algorithms for future dynamic nonlinear optimization with estimation on Hessian matrix inversion. IEEE Trans. Syst. Man Cybernet. Syst. 51, 2611–2623. 10.1109/TSMC.2019.2916892

[B28] Zhang W. Liu L. Song G. (2020). Design of bionic knee joint mechanism based on tensegrity structure. Eng. Struct. 44, 98–104. 10.16578/j.issn.1004.2539.2020.12.015

[B29] Zhao H. Xu X. (2011). Torque parameters of human knee joint. J. Clin. Rehabil. Tissue Eng. Res. 15, 705–708. 10.3969/j.issn.1673-8225.2011.04.033

